# Reduction in Radiation Exposure of CT Perfusion by Optimized Imaging Timing Using Temporal Information of the Preceding CT Angiography of the Carotid Artery in the Stroke Protocol

**DOI:** 10.3390/diagnostics12112853

**Published:** 2022-11-18

**Authors:** Zsuzsanna Deak, Lara Schuettoff, Ann-Kathrin Lohse, Matthias Fabritius, Paul Reidler, Robert Forbrig, Wolfgang Kunz, Konstantin Dimitriadis, Jens Ricke, Bastian Sabel

**Affiliations:** 1 Imaging Urania, Laurenzerberg 2, 1010 Vienna, Austria; 2 Department of Radiology, University Hospital of Munich (LMU), Marchioninistr. 15, 81377 Munich, Germany; 3 Department of Neuroradiology, University Hospital of Munich (LMU), Marchioninistr. 15, 81377 Munich, Germany; 4 Department of Neurology, University Hospital of Munich (LMU), Marchioninistr. 15, 81377 Munich, Germany

**Keywords:** stroke, CT perfusion, carotid CT angiography, optimized imaging timing, dose reduction, personalized imaging

## Abstract

(1) Background: CT perfusion (CTP) is a fast, robust and widely available but dose-exposing imaging technique for infarct core and penumbra detection. Carotid CT angiography (CTA) can precede CTP in the stroke protocol. Temporal information of the bolus tracking series of CTA could allow for better timing and a decreased number of scans in CTP, resulting in less radiation exposure, if the shortening of CTP does not alter the calculated infarct core and penumbra or the resulting perfusion maps, which are essential for further treatment decisions. (2) Methods: 66 consecutive patients with ischemic stroke proven by follow-up imaging or endovascular intervention were included in this retrospective study approved by the local ethics committee. In each case, six simulated, stepwise shortened CTP examinations were compared with the original data regarding the perfusion maps, infarct core, penumbra and endovascular treatment decision. (3) Results: In simulated CTPs with 26, 28 and 30 scans, the infarct core, penumbra and PRR values were equivalent, and the resulting clinical decision was identical to the original CTP. (4) Conclusions: The temporal information of the bolus tracking series of the carotid CTA can allow for better timing and a lower radiation exposure by eliminating unnecessary scans in CTP.

## 1. Introduction

Especially in the early phase, reliable and fast diagnosis is crucial to improve the outcome of thrombolysis in the new era of advanced stroke treatment. Although magnetic resonance imaging (MRI) is the most sensitive method to detect the tissue at risk, CT perfusion (CTP) imaging, a fast and widely available imaging modality, is also able to differentiate the infarct core from the penumbra in patients with suspected stroke. The current CTP acquisition, which covers almost the whole brain, operates in cine mode and scans the first pass, peak and wash-out of the intravenous administered contrast agent in brain tissue. CTP maps depicting cerebral blood volume, cerebral blood flow, mean transit time, time to drain and time to maximum can be generated to differentiate the ischemic core from penumbra, and even automated volume calculations are available [1,2,3,4,5]. CTP is a very dose-intensive imaging method. The average radiation exposure of 2.7 mGy for carotid and cerebral CT angiographies is considerably lower than the radiation exposure during perfusion measurements of the brain of about 100–190 mGy. Stochastic radiation risks decrease substantially with age and are markedly higher for females than for males. To balance the diagnostic needs and patient protection, CT perfusions studies have to be strictly justified and carefully optimized [6,7,8]. Since it contributes considerably to the CT-associated radiation exposure of patients, several studies focused on methods for reducing the dose—for example, by lowering the tube voltage or tube current, lowering the acquisition frequency or prolonging the time interval between scans [9].

For patients who otherwise meet the criteria for endovascular therapy, a noninvasive intracranial vascular study is recommended during the initial imaging evaluation of the acute stroke patient, and it is reasonable to proceed with CTA, if indicated, in patients with suspected intracranial large vessel occlusion, as well as to apply CT perfusion to determinate collateral flow status and to determine the eligibility for mechanical thrombectomy [10].

Carotid CT angiography can precede CTP in the diagnostic CT algorithm in stroke patients, and the order of CT angiography and CTP does not significantly affect the quantitative parameters of CTP [11]. The timing of image acquisition after intravenous contrast administration is of great importance to obtain optimal contrast in the intra- and extracranial arteries in the CT angiography. Typically, an aortic bolus tracking is performed, and image acquisition begins when intraluminal enhancement of the ascending aorta exceeds the threshold value. If CT angiography precedes CTP, the arrival of the contrast bolus in the ascending aorta is known and could be interpreted as a test bolus. Using this information, a better timing of the CTP data acquisition could be achieved.

In our study, we used the temporal information of the bolus tracking series of a preceding CT angiography to investigate whether optimal timing of the CTP has the potential to reduce the number of scans applied and whether shortening the CTP affects the automatically calculated infarct core and penumbra. In addition, we investigated whether altering the number of CTP scans in this manner significantly affects the resulting maps as well as influences the resulting clinical decision pathway. These are critical to the decision to choose interventional or conventional lysis therapy.

In the current literature, there is no comparable study using the bolus tracking series of the carotid CT angiography to assess the optimal timing for CT perfusion of the brain.

## 2. Materials and Methods

### 2.1. Patient Population

Our study was conducted in accordance with the Declaration of Helsinki, approved by the local ethical committee and registered with the following ID: DRKS00023941 in the German Clinical Trials Register. The imaging data of all included patients were retrospectively collected and anonymized.

**Figure 1:**
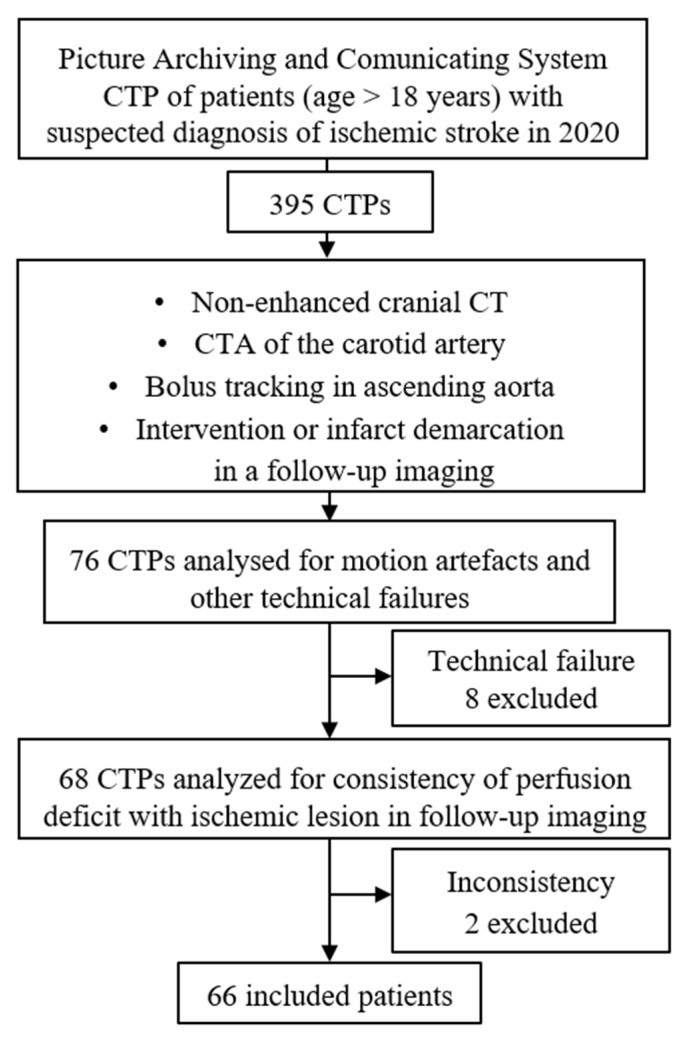
Flow chart of patient recruitment.

Patients with a suspected diagnosis of ischemic stroke who received CTP from 1 January to 31 December 2020 were searched for in our institutional Picture Archiving and Communicating System. Patients with a perfusion deficit had to meet the following inclusion criteria: age over 18 years, institutional standard imaging protocol including non-enhanced cranial CT, CT angiography of the carotid arteries and CTP, follow-up imaging or subsequent intervention and archived scans of the bolus tracking in the ascending aorta. Patients with perfusion disturbances in the initial CTP imaging but without any evidence of an ischemic event in the follow-up imaging were not included in the study group. A total of 395 patients with suspected ischemic stroke received the institutional standard protocol during this period. A total of 76 patients with available bolus tracking records and perfusion deficits who had infarct demarcation in the follow-up imaging or underwent mechanical intravascular procedures because of cerebral artery occlusion were included in our study. A total of 10 patients were excluded: 2 due to the inconsistency of the perfusion deficit with the infarct lesion in the follow-up imaging, 2 due to an invalid bolus tracking dataset (technical problem) and 6 due to an invalid CTP dataset (motion artifacts). The total of 66 patients (33 males, 33 females; mean age ± standard deviation: 71.3 ± 15.2 years, ranging from 28 to 96) constituted our study group. Patient recruitment is shown as a flow chart in Figure 1.

### 2.2. CT Examination Protocol

Imaging was performed on a 192-detector-row dual-source-CT scanner (Somatom Force; Siemens Healthineers, Erlangen, Germany) according to the vendor’s recommendations. The following optimized scan parameters were applied for CTP in each patient: whole brain coverage with a scan length of 294 mm, applying a tube voltage of 70 kV, a fixed tube current of 250 mAs and a rotation time of 0.25 s and acquiring 32 scans with a time interval of 1.5 s between two scans. The contrast agent was administered in the antecubital vein as a bolus of 40 mL, with 300 mg iodine per mL (Imeron; Bracco Imaging Deutschland, Konstanz, Germany) at a flow rate of 6 mL per second, followed by a saline bolus of 50 mL. The scan phase began 8 s after the start of the contrast agent injection. For the CT angiography of the carotid arteries, the contrast agent was administered as a bolus of 40 mL at a flow of 5 mL per second, and the first bolus tracking scan began 7 s after injection. The tracking scans were performed at the level of the ascending aorta, with a frequency of 1 per second. The scanning period of the CT angiography began as the intraluminal enhancement of the aorta reached the threshold CT attenuation value of 100 HU.

### 2.3. Image Processing and Volumetry

After image acquisition, all CTP datasets: 32 scans—each of them operating with 116 slices of 1.5 mm—were reconstructed, anonymized and transferred to a dedicated image processing software. For all datasets, we performed perfusion analysis with the vendor-given Syngo Volume Perfusion CT Neuro software (Syngo.Via VB40A 2019; Siemens Healthineers, Erlangen, Germany). The infarct core and penumbra were automatically calculated by applying the same software using the vendor-given fixed specification for cerebral blood volume and cerebral blood flow threshold values: cerebral blood volume < 1.2 mL/100 mL and cerebral blood flow < 35.1 mL/100 mL [12,13,14]. The arterial input region was selected from the vessels of the anterior cerebral arteries, and the venous input was located in the superior sagittal sinus. A set of 31 color-coded slices were calculated for each of the five CTP maps (cerebral blood volume, cerebral blood flow, mean transit time, time to drain and time to maximum). Perfusion deficits were analyzed; the volumetric data of the infarct core [mL] and penumbra [mL] and the data of the automatically assessed Potential Recuperation Ratio (PRR, [%]) were extracted [15].

### 2.4. Study Design

In each patient, simulated and gradually shortened CTP datasets were generated using the temporal information of the contrast agent arrival in the ascending aorta. The temporal information was extracted from the corresponding bolus tracking scans. In a preliminary study of 50 patients receiving a valid CTP, enhancement curves and aortic bolus arrival were analyzed to compare the delay of the contrast agent arrival in the ascending aorta and in the intracranial arteries. Considering these results, an exact time interval was identified to define the first scan of the CTP dataset in the simulated CTP (sCTP) examinations of our patient study group. This interval was constant for each patient and was considered in each patient. It was added to the personalized time information we retrieved from the corresponding data of the aortal bolus tracking series in each patient, and it was respected in all simulated datasets to identify the first scan. After the first scan of the sCTPs was selected and tagged, we generated shortened scan series: the 32 scans of the oCTPs were gradually decreased to 30 scans (sCTP_30_), 28 scans (sCTP_28_), 26 scans (sCTP_26_), 24 scans (sCTP_24_), 22 scans (sCTP_22_) and 20 scans (sCTP_20_), resulting in six additional sCTP datasets in each patient.

In the next step, all of the six sCTP examination (66 × 6 = 396) datasets were reloaded into Syngo.Via, and the infarct core, penumbra and PRR (potential recuperation ratio) were automatically calculated for each patient of the study group.

PRR values of the simulated datasets (sCTPs) were compared to PRR values of the original imaging data (oCTPs) and analyzed regarding discrepancy.

In all perfusion CT examinations including original imaging data and simulated datasets, the clinical decision pathway concerning the differentiation between the “target mismatch profile” (volume of infarct core ≤ 50 mL; volume of mismatch > 15 mL; volume of mismatch/volume of infarct core ≥ 1.8) and the “malignant profile” was examined according to previous studies investigating stroke evolution and the effect of intravenous thrombolysis with or without mechanical thrombectomy [16,17,18,19].

The data of radiation exposure were extracted from the clinical dose-monitoring software (Radimetrics, Bayer, Germany, Leverkusen).

The relationship between the delay of the aortal bolus arrival and the chronological data of the wash-in and wash-out phases was analyzed.

### 2.5. Statistical Analysis

A paired sample t-test was used to investigate the equivalence of the calculated values for the infarct and penumbra and the PRR values between oCTP and the six sCTPs. The level of significance was set at a *p*-value of 0.05. With the aid of Bonferroni correction, the confidence interval was adjusted because of multiple comparisons, and the significance level was set at 0.003 (0.05/18 = 0.00278).

The linear relationship between the aortal bolus arrival, the arterial inflow peak, the venous inflow peak, the end point of the wash-out phase and the duration of the complete perfusion curve covering both the wash-in and the wash-out phase was investigated by calculating the Pearson product–moment correlation coefficient.

The statistical calculations were performed with a statistical software (SPSS 22.0, IBM Corporation, Armonk, NY, USA).

## 3. Results

The mean value and the standard deviation for the infarct core volume and penumbra in the standard CTP protocol were 35.71 ± 34.83 mL (ranged from 6.4 mL to 218.59 mL) and 131.37 ± 59.71 mL (ranged from 45.45 mL to 338.61 mL), respectively. The radiation exposure of the standard protocol operating with 32 scans was 196.13 mGy (computed tomography dose index) and 2908.3 mGycm (dose length product).

The results of the volumetric calculations of the shortened simulated CTPs, including *p*-values and radiation exposure, are shown in Table 1.

In the comparative analysis of the PRR values of standard and simulated CTPs, the percentages of the simulated examinations, which present a deviation not exceeding 1%, 3%, 5% and 10%, are given in Table 2.

In standard CTP, a “target mismatch” profile was identified in 55 of the 66 patients. We detected a “malignant” profile in nine patients because of a large infarct core (>50 mL) and in two patients because of an insufficient mismatch (ratio < 1.8). The number of patients with a “malignant” profile was higher in some of the simulated CTPs: 21 (sCTP_20_), 19 (sCTP_22_) and 15 (sCTP_24_). In the simulated CTPs with 26, 28 and 30 scans, the patients with a “target mismatch” profile were identical.

The mean value ± standard deviation for the aortal bolus delay was 11.92 ± 3.33 s, and it ranged from 7.0 s to 19.0 s. The mean value ± standard deviation for the arterial inflow peak was 22.83 ± 4.81 s, and it ranged from 14.0 s to 38.0 s. The mean value ± standard deviation for the venous inflow peak was 27.81 ± 5.75 s, and it ranged from 17.0 s to 42.5 s. The mean value ± standard deviation for the end point of the wash-out phase was 41.01 ± 7.60 s, and it ranged from 7.0 s to 54.5 s. The mean value ± standard deviation for the duration of the complete perfusion curve was 30.13 ± 5.13 s, and it ranged from 19.0 s to 39.0 s.

The correlation analysis showed a strong positive correlation of the aortal bolus arrival with all evaluated parameters of the perfusion curve (arterial inflow peak: 0.81520; *p* ˂ 0.00001, venous inflow peak: 0.81510; *p* ˂ 0.00001, end point of the wash-out phase: 0.8623; *p* ˂ 0.00001, duration of complete perfusion: 0.8534; *p* ˂ 0.00001).

## 4. Discussion

CT perfusion is a high-dose exposure imaging technique requiring optimization. With the aid of the temporal information of the aortal bolus arrival, we could effectively reduce the radiation level of CTP without a loss of substantial information.

Acute ischemic stroke, with a high global incidence, is commonly responsible for severe disability in developed nations. Thrombolysis in the acute setting including endovascular intervention is essential and requires fast and reliable diagnostics [20].

After MRI, CTP is the most sensitive diagnostic tool to offer reliable identification and assessment of the infarct core and ischemic penumbra, and it is well integrated into CT-based stroke protocols [21].

However, the recommended temporal resolution of 1 or 2 scans per second, which provides a precise plotting of time-attenuation curves, is associated with a substantial radiation exposure. For this reason, many publications have addressed the dose reduction of CTP—for example, by adjusting the tube current, temporal resolution or total scan duration, by modifying the chaser bolus volume and by using modern reconstruction algorithms to optimize imaging and to fulfil the ALARA (As Low As Reasonably Achievable) principle [22,23,24,25,26].

**Figure 2:**
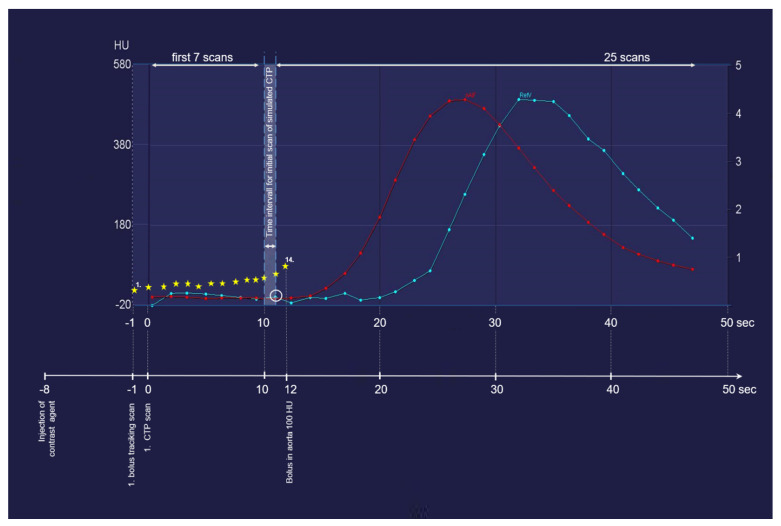
With the aid of an example, this figure explains the method of identifying the first scan of the simulated data sets. The CT attenuation values of the bolus tracking scan are marked with *. The *x* axis indicates the time passed after the contrast agent injection. The *y* axis represents the corresponding attenuation values. In the simulated CTP examinations, the initial scan was set to at least 1 s but not more than 2 s before the time at which the contrast agent bolus approached the threshold value of 100 HU in the ascending aorta. This means that if the contrast agent reached the threshold value (for example, 20 s after injection), the first CTP scan should be chosen around 18 s after injection, corresponding to the 8th scan acquired at 19 s after the contrast agent injection in this case. nAIF: arterial inflow peak; RefV: venous inflow peak.

Given that CT angiography of the carotid artery, which precedes CTP in the diagnostic CT algorithm, has no influence on CTP results [11], the bolus arrival in the aorta could be considered as a test bolus that provides information for the optimal timing of CTP. In the simulated CTP examinations in our study, the initial scan was set to at least 1 s but not more than 2 s before the time at which the contrast agent bolus approached the threshold value of 100 HU in the ascending aorta. This means that if the contrast agent reached the threshold value (for example, 20 s after injection), we declared the first CTP scan 18 s after injection—in this case, the 8th scan acquired 19 s after the contrast agent injection—to be the initial CTP scan. The relationship and temporal data of the attenuation curves of the bolus tracking series, arterial inflow and venous inflow are visualized in Figure 2.

**Figure 3:**
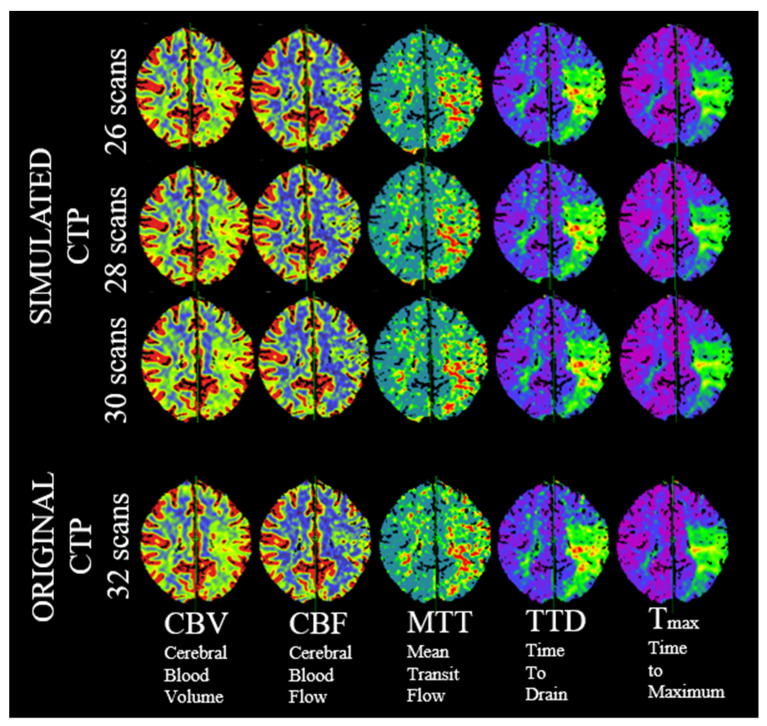
Visually identical perfusion maps of the original and the simulated examinations operating with 30, 28 or 26 scans.

Using this method, we were able to generate personalized shortened CTPs with the same diagnostic quality, allowing for a dose reduction of at least 18.75% in all patients without a loss of image information and without changing the clinical decision based on the imaging results in our study. The calculated CTP maps were also visually identical in all cases with comparable volumetric results. An example is presented in Figure 3.

**Figure 4:**
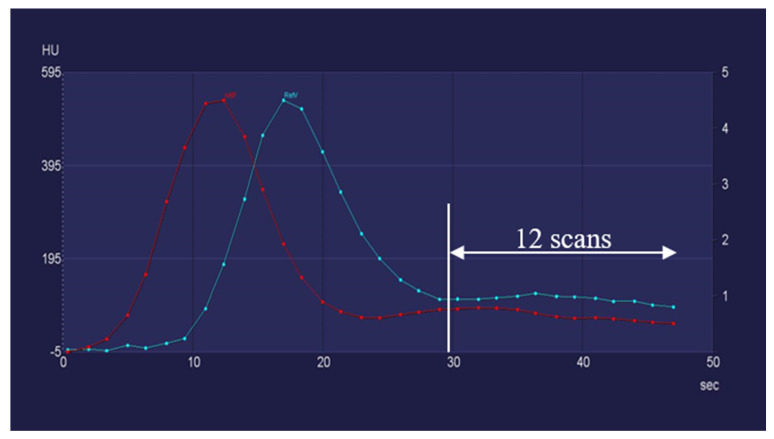
In patients with a fast bolus arrival, the number of scans could be decreased to 20.

Furthermore, PRR values showed no more than 5% deviation in 75.8% of sCTP_24_ (50 patients) and 62.1% of sCTP_22_ (41 patients) compared with the oCTP series. These results suggest that, in certain cases, a further dose reduction could be achieved by lowering the number of scans to 24 or even to 20. Underlying this, 10.6% of the sCTP_20_ (7 patients) had PRR values that were more than 99% identical to oCTP series. These results indicate that, in these cases, the number of scans could be decreased to 20 without any impairment of the diagnostic quality, which corresponded to a 28.5 s-long scanning window and a dose reduction of 37.5%. The duration of the wash-in and wash-out phase took less than 24 s in these patients, and they also had a very fast aortal bolus arrival as well as an early arterial and venous inflow peak. The flow curves of a corresponding patient are visualized in Figure 4.

Our correlation analysis indicates a strong relationship between a fast aortal bolus arrival and a fast wash-in phase and an early arterial inflow peak and a fast wash-out phase.

Considering this information, personalized CTP protocols with an optimized number of perfusion scans as well as dose-optimized CT examinations could be easily available and should be applied in all suitable patients.

**Figure 5:**
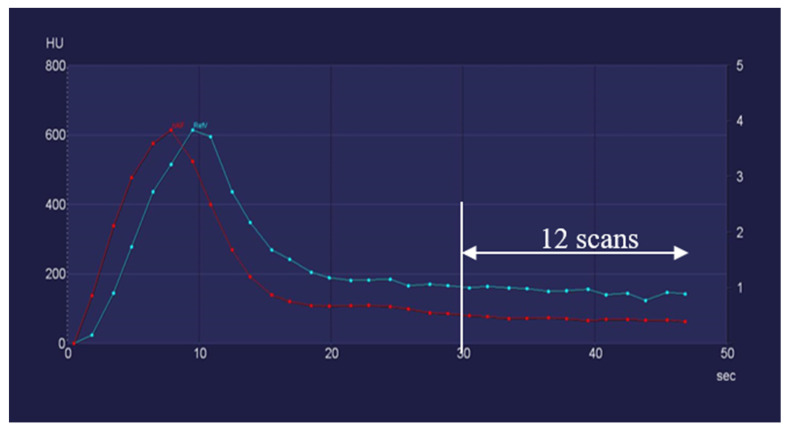
A shift in the ascending arterial inflow curve closer to the venous inflow curve was detected in the case of a very early contrast arrival in the ascending aorta.

On the other hand, bolus tracking in 4 patients (6.1%) revealed a very early contrast arrival (enhancement well above 100 HU in the first bolus tracking scan) in the ascending aorta, resulting in a shift in the ascending arterial inflow curve closer to the venous inflow curve in the original CTP and indicating an unwelcome delay of the original CTP, suggesting a probable loss of information. The flow curves of a corresponding patient are visualized in Figure 5.

The classic CTP protocol recommended by Wintermark et al., applying a 40 s scanning window starting 7 s after the contrast injection, has similar characteristics to the protocol we used, with a delay of 8 s, a scanning window of 46.5 s and a scan interval of 1.5 s [25,26].

Corcuera-Solano et al. advocated for the use of adapted variable and extended sampling (3 passes every 3 s intended for the baseline before arrival, 13 passes every 1.5 s for the rise and fall of enhancement and 3 passes every 7.5 s to complete and extend the sampling window to 51 s) to improve quality and to reduce radiation exposure to 1588 mGycm at 70 kVp. However, Kasasbeh et al. found that an acquisition duration of 65 s or longer is required [27,28].

**Figure 6:**
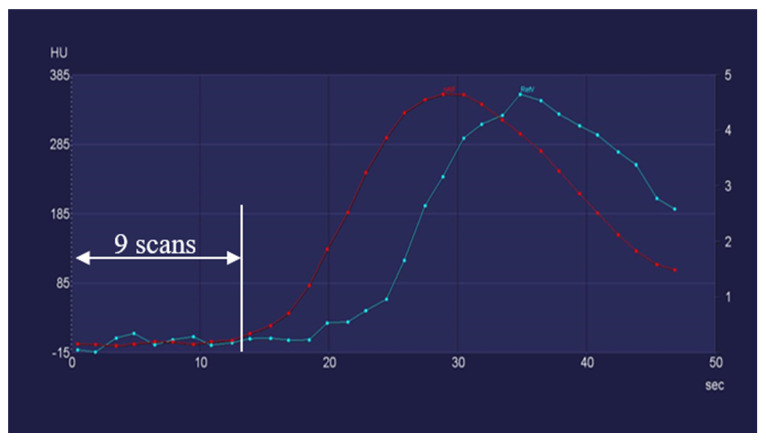
In the case of a slow bolus arrival, the descending slope of the arterial and venous inflow curves becomes truncated.

The lack of optimal timing can significantly impede the efficiency of this scanning technique. Due to the variation in cardiac output among patients (for example, the aortal bolus arrival ranged from 7 to 19 s in our study group), the overall duration of the entire contrast circle, including the arterial arrival and venous return, might not be appropriately captured if it does not fit within the scan window. In this case, patients with a delayed bolus arrival may have a truncated descending slope of the venous wash-out curve; we observed this phenomenon in seven patients (10.6%) in our study. The flow curves of a corresponding patient are visualized in Figure 6.

These findings indicate the urgent need for proper and personally adapted timing of CTP imaging instead of the use of a fixed delay to receive high-quality perfusion data in all patients. Moreover, in patients with a very slow bolus arrival, a prolonged scanning interval instead of 1 or 2 s could be considered to improve the quality of the data acquisition.

However, the manual adjustment of the timing of the imaging or the imaging parameter can be highly operator-dependent. The integration of the temporal information of the bolus tracking series of the carotid CT angiography into the CT perfusion protocol by the vendor might reduce the operator-dependent character of this method.

We acknowledge the limitations of our study. The injection rates of contrast agents were similar for CT angiography (5 mL/s) and CTP (6 mL/s) but not completely identical. Because of the automated software-based calculation method, our results are quite reproducible within our study but not comparable to the results of other software that use different parameters for infarct core and penumbra identification (e.g., RAPID; iSchemaView, Menlo Park, CA, USA).

## 5. Conclusions

In conclusion, the timing information of the bolus tracking of previous carotid CT angiography can be used in the stroke protocol to reduce radiation exposure by avoiding unnecessary scans. In addition, the information from bolus tracking allows for the better timing of CTP, which could lead to better outcomes in certain cases by adjusted timing.

## Figures and Tables

**Table 1:** The data of the volumetric calculations of the shortened simulated CTPs are given as the mean value, standard deviation, minimum and maximum, including *p*-values and the data of radiation exposure as the CT dose index (CTDI). Significant *p* values are marked with *.

		Simulated CTP_30_		Simulated CTP_28_		Simulated CTP_26_		Simulated CTP_24_		Simulated CTP_22_		Simulated CTP_20_	
	Number of Scans	30		28		26		24		22		20	
	CTDI	183.9 mGy		171.6 mGy		159.4 mGy		147.1 mGy		134.8 mGy		122.6 mGy	
**Infarct core** **(mL)**	Mean	33.01	*p* value = 0.1930	37.52	*p* value = 0.8880	36.97	*p* value = 0.0564	38.58	*p* value = 0.0001 *	39.03	*p* value = 0.0024 *	41.57	*p* value < 0.0001 *
	Standard deviation	29.48		36.65		36.34		36.79		38.07		38.19	
	Minimum	8.31		6.34		6.16		6.04		3.49		6.00	
	Maximum	174.75		219.21		222.55		226.50		228.6		225.87	
**Penumbra** **(mL)**	Mean	118.86	*p* value = 0.1215	132.68	*p* value = 0.3609	131.64	*p* value = 0.0508	119.82	*p* value < 0.0001 *	109.86	*p* value < 0.0001 *	102.96	*p* value < 0.0001 *
	Standard deviation	59.86		62.37		57.85		54.23		52.55		49.29	
	Minimum	46.17		43.39		43.84		40.73		11.82		2.26	
	Maximum	174.75		326.08		317.44		296.27		175.05		245.10	
**Potential Recuperation** **Ratio**	Mean	78.0%	*p* value = 0.7369	77.0%	*p* value = 0.7071	78.7%	*p* value = 0.7805	76.6%	*p* value < 0.0001 *	74.6%	*p* value < 0.0001	71.2%	*p* value < 0.0001 *
	Standard deviation	13.2%		16.7%		13.3%		13.6%		14.2%		17.8%	
	Minimum	35.5%		0.8%		33.7%		28.9%		27.3%		8.8%	
	Maximum	95.4%		96.0%		96.5%		93.6%		92.9%		92.8%	

**Table 2:** The results of the comparative analysis of the PRR values of standard and simulated CTPs are given as the percentages of the simulated examinations, which present a deviation not exceeding 1%, 3%, 5% and 10%.

		sCTP_30_	sCTP_28_	sCTP_26_	sCTP_24_	sCTP_22_	sCTP_20_
Number of Scans		30	28	26	24	22	20
Percentage of patients with a difference in PRR value of	≤1%	84.5%	58.5%	55.4%	24.2%	15.2%	10.6%
	≤3%	96.9%	86.2%	84.8%	50.0%	36.4%	22.7%
	≤5%	100.0%	100.0%	100.0%	75.8%	62.1%	40.9%
	≤10%	100.0%	100.0%	100.0%	97.0%	78.8%	66.7%

## Abbreviations

CT	computed tomography
CTP	CT perfusion
MRI	magnetic resonance imaging
PRR	potential recuperation ration
oCTP	original CTP
sCTP	simulated CTP
